# Experimental Study and ANN Development for Modeling Tensile and Surface Quality of Fiber-Reinforced Nylon Composites

**DOI:** 10.3390/polym17111528

**Published:** 2025-05-30

**Authors:** Osman Ulkir, Fatma Kuncan, Fatma Didem Alay

**Affiliations:** 1 Department of Electric and Energy, Mus Alparslan University, Mus 49250, Turkey; 2 Department of Computer Engineering, Siirt University, Siirt 56100, Turkey; fatmakuncan@siirt.edu.tr; 3 Department of Computer Engineering, Harran University, Sanliurfa 63300, Turkey; fdidemogretmen@harran.edu.tr

**Keywords:** fused deposition modelling, artificial neural networks, carbon fiber-reinforced nylon composites, tensile strength, surface roughness, machine learning

## Abstract

Additive manufacturing (AM) is gaining widespread adoption in the manufacturing industry due to its capability to fabricate intricate and high-performance components. In parallel, the increasing emphasis on functional materials in AM has highlighted the critical need for accurate prediction of the mechanical behavior of composite systems. This study experimentally investigates the tensile strength and surface quality of carbon fiber-reinforced nylon composites (PA12-CF) fabricated via fused deposition modeling (FDM) and models their behavior using artificial neural networks (ANNs). A Taguchi L27 orthogonal array was employed to design experiments involving five critical printing parameters: layer thickness (100, 200, and 300 µm), infill pattern (gyroid, honeycomb, and triangles), nozzle temperature (250, 270, and 290 °C), printing speed (50, 100, and 150 mm/s), and infill density (30, 60, and 90%). An analysis of variance (ANOVA) revealed that the infill density had the most significant influence on the resulting tensile strength, contributing 53.47% of the variation, with the strength increasing substantially at higher densities. In contrast, the layer thickness was the dominant factor in determining surface roughness, accounting for 53.84% of the variation, with thinner layers yielding smoother surfaces. Mechanistically, a higher infill density enhances the internal structural integrity of the parts, leading to an improved load-bearing capacity, while thinner layers improve the interlayer adhesion and surface finish. The highest tensile strength achieved was 69.65 MPa, and the lowest surface roughness recorded was 9.18 µm. An ANN model was developed to predict both the tensile strength and surface roughness based on the input parameters. Its performance was compared with that of three other machine learning (ML) algorithms: support vector regression (SVR), random forest regression (RFR), and XGBoost. The ANN model exhibited superior predictive accuracy, with a coefficient of determination (R^2^ > 0.9912) and a mean validation error below 0.41% for both outputs. These findings demonstrate the effectiveness of ANNs in modeling the complex relationships between FDM parameters and composite properties and highlight the significant potential of integrating ML and statistical analysis to optimize the design and manufacturing of high-performance AM fiber-reinforced composites.

## 1. Introduction

AM has become a transformative technology in modern industry due to its ability to produce complex geometries, reduce material waste, and enable rapid prototyping [1,2]. It offers significant advantages over conventional manufacturing methods, particularly for customized and low-volume production. AM is widely used across various fields, including the aerospace, automotive, biomedical, and consumer electronics fields [3,4,5]. Among the numerous AM techniques, FDM has emerged as one of the most prominent methods for fabricating polymer-based components. Due to its low cost, ease of use, and adaptability, FDM has been widely adopted in industrial applications such as medical devices, robotics, and tooling, where fast prototyping and design flexibility are essential [6,7].

In the FDM process, a wide range of thermoplastic materials can be used, including acrylonitrile butadiene styrene (ABS), polylactic acid (PLA), polyethylene terephthalate glycol (PETG), and engineering-grade polymers such as Polyamide 12 (PA12) [8,9,10]. Furthermore, composite materials reinforced with short or continuous fibers have gained increasing attention due to their enhanced mechanical performance [11]. In this study, carbon fiber-reinforced nylon (PA12-CF) is selected as the filament material due to its high strength/weight ratio, dimensional stability, and thermal resistance, which make it particularly suitable for use in industrial applications such as automotive parts, aerospace brackets, and functional end-use components [12,13]. Among fiber-reinforced thermoplastics, both PA6-CF and PA12-CF are widely studied due to their excellent mechanical properties and thermal stability. However, important differences exist between these two matrix materials that affect their performance and processability in additive manufacturing applications. PA6-CF typically exhibits higher stiffness and thermal resistance due to its higher degree of crystallinity and lower moisture absorption at room temperature [14]. However, PA6 is more hygroscopic and can absorb more moisture over time, which may negatively impact its dimensional stability and interfacial bonding in humid environments. In contrast, PA12-CF offers lower moisture absorption, better chemical resistance, and superior dimensional stability, which makes it more suitable for applications that require long-term durability and precision under varying environmental conditions. In terms of processability, PA12 has a lower melting point (~178–180 °C) compared to PA6 (~220–225 °C), which allows for easier fusion during the FDM process and reduces the risk of thermal degradation. Moreover, PA12 exhibits lower water uptake and improved flexibility, which is advantageous for minimizing warpage and improving interlayer adhesion in 3D printing. Therefore, although PA6-CF is commonly used in thermoplastic pultrusion and structural applications, PA12-CF is increasingly preferred in FDM-based additive manufacturing for its balanced mechanical performance, thermal stability, and superior printability. However, the mechanical and surface properties of FDM-printed parts are highly sensitive to printing parameters such as the layer height, printing speed, and nozzle temperature. Therefore, the careful selection and optimization of these parameters is essential to ensuring improved performance, which underscores the importance of analyzing these printing parameters in detail.

The development of AI-driven predictive models is crucial to achieving optimal mechanical properties in FDM-printed composite parts, particularly when fiber-reinforced thermoplastics are used [15,16,17]. Artificial neural networks (ANNs) and other machine learning (ML) algorithms are powerful tools for predicting process outcomes and optimizing process parameters without extensive physical testing [18,19,20]. These models can capture the complex nonlinear relationships between input variables and output responses, enabling more efficient and accurate control over the printing process. As such, ML-based approaches are becoming increasingly important in the field of AM, particularly in the performance-driven design and manufacturing of composite materials [21,22].

Despite the growing interest in using ML to predict the behavior of FDM-printed fiber-reinforced composites, studies specifically targeting the mechanical and surface properties of PA12-CF materials remain limited [23,24,25,26,27]. For instance, in the study by Fetecau et al. [28], an ANN was developed to predict the Young’s modulus and tensile strength of 3D-printed PA12 and its carbon- and glass fiber-reinforced composites. The model was based on two key input parameters: infill density and printing orientation. Both single-target and dual-output ANN architectures were investigated using the Levenberg–Marquardt algorithm, which achieved prediction errors below 5%. However, this work focused solely on mechanical properties and did not consider the surface quality, nor did it compare the ANN’s performance against that of other ML algorithms. In another recent study by Ahlawat et al. [29], a hybrid optimization approach that combined an ANN with a multi-objective genetic algorithm (MOGA) was employed to optimize the tensile, compression, impact, and wear properties of carbon fiber-reinforced nylon composites. FDM was used, and a broad set of mechanical tests was performed. The ANN model was trained on experimental data, and the MOGA was used to identify optimal combinations of the process parameters that maximize performance. Although this study provides a comprehensive multi-property optimization, it does not include surface roughness as a response variable and lacks a comparative analysis with alternative ML models. A third study by Belei et al. [30] focused on optimizing the tensile strength of short carbon fiber-reinforced polyamide parts via fused filament fabrication, using both design of experiments (DoE) and machine learning techniques. A 2^k full factorial DoE was combined with a multiple polynomial regression model and an ML-based algorithm using scikit-learn. The study found that the layer height and bed temperature were the most influential factors that affected the obtained tensile properties. While it effectively demonstrated the role of individual process parameters, the study was limited to a small parameter set and did not explore multiple ML algorithm performances or surface characteristics. While previous studies have explored the use of ANNs or other ML techniques to predict specific mechanical properties of FDM-printed fiber-reinforced composites, they often suffer from three major limitations: (1) a narrow focus on a single output variable (typically tensile strength), (2) a limited number of printing parameters considered, and (3) a lack of comparative validation against alternative ML models. Moreover, surface quality—a critical factor in functional applications—has largely been neglected in ANN-based modeling frameworks. This study addresses these gaps by proposing a dual-output predictive model based on an ANN that is capable of simultaneously estimating the tensile strength and surface roughness of composites using five key FDM parameters. The model is trained on a systematically designed experimental dataset using a Taguchi L27 orthogonal array, ensuring wide parameter coverage and statistical robustness. Furthermore, the ANN model is benchmarked against three commonly used ML algorithms—SVR, RFR, and XGBoost—under identical conditions to provide a comprehensive comparative evaluation. The novelty of this work lies not only in the multi-output capability of the constructed ANN model but also in its integration with a rigorous experimental design and performance benchmarking. This hybrid approach enables both accurate prediction and deeper understanding of the complex, nonlinear relationships between FDM parameters and composite performance. As such, this study contributes a more generalizable, scalable, and application-oriented machine learning framework to the additive manufacturing literature. It is expected to support real-time process optimization, reduce trial-and-error in production settings, and facilitate the development of intelligent manufacturing systems for fiber-reinforced thermoplastics.

This study focuses on the experimental investigation and ML-based modeling of the tensile strength and surface roughness of PA12-CF composites printed using FDM technology. Five key printing parameters—the layer thickness, infill pattern, nozzle temperature, printing speed, and infill density—were varied using a Taguchi L27 orthogonal array to design the experiments. Following mechanical testing and surface analysis, an ANN model was developed and validated, and its prediction performance was compared with that of SVR, RFR, and XGBoost models. The results highlight the strong predictive capability of the ANN, which obtained an R^2^ value exceeding 0.9912 and a mean validation error below 0.41%. This study demonstrates the potential of integrating experimental design with advanced ML techniques to enhance process optimization and property prediction in FDM of fiber-reinforced composites.

## 2. Materials and Methods

### 2.1. Material

In this study, the material selected for the fabrication of test specimens is Flashforge PA12-CF, a carbon fiber-reinforced polyamide 12 composite filament. This material combines the ductility and chemical resistance of nylon with the enhanced mechanical strength and rigidity provided by short carbon fibers. The filament is specifically engineered for high-performance FDM applications, offering excellent dimensional stability, reduced warping, and improved surface finish even on unheated or low-temperature print beds. The composite filament consists primarily of a PA12 nylon matrix and short chopped carbon fibers, and has an approximate fiber content of 15–20% by weight. The PA12 resin exhibits a tensile strength of ~45–55 MPa, an elongation at break of 150–200%, and a melting temperature in the range of 178–180 °C. It is known for its low water absorption, good fatigue resistance, and inherent flexibility. The short carbon fibers used as reinforcement typically have a tensile strength exceeding 3.5 GPa, a Young’s modulus of ~230 GPa, and a diameter of 7–10 µm. These fibers significantly enhance the stiffness and strength of the composite, while also contributing to its dimensional stability and thermal conductivity. PA12-CF is capable of withstanding continuous use up to 120 °C without exhibiting significant degradation, which makes it suitable for structural applications in demanding environments. Mechanically, the material exhibits superior performance compared to standard nylon filaments. It features a tensile strength of at least 50 MPa, a flexural strength above 60 MPa, and a notched Izod impact strength exceeding 105 J/m, according to ASTM D256. Additionally, the filament offers high ductility, with an elongation at break of over 150%, which allows it to absorb mechanical shocks without sudden failure. Its thermal decomposition begins above 100 °C and its density is approximately 1.4 g/cm^3^, which indicate that it has a relatively lightweight yet robust structure. Furthermore, the formulation of this material is optimized to reduce moisture absorption, providing four-times-slower saturation than conventional PA6 filaments, which greatly enhances its long-term stability and print reliability. Due to the abrasive nature of the carbon fibers in the composite, the use of hardened steel or ruby nozzles is recommended to prevent wear during printing. The material’s printing profile includes an extrusion temperature range of 270–290 °C, a bed temperature between 80 and 100 °C, and a print speed of 40–60 mm/s. In summary, the PA12-CF filament used in this study combines the flexibility and chemical resistance of PA12 matrix with the high strength and stiffness of carbon fibers, which results in a composite material suitable for use in high-performance functional components for industrial, automotive, aerospace, and robotic applications.

### 2.2. FDM Fabrication Process and Design of Experiments

**Figure 1:**
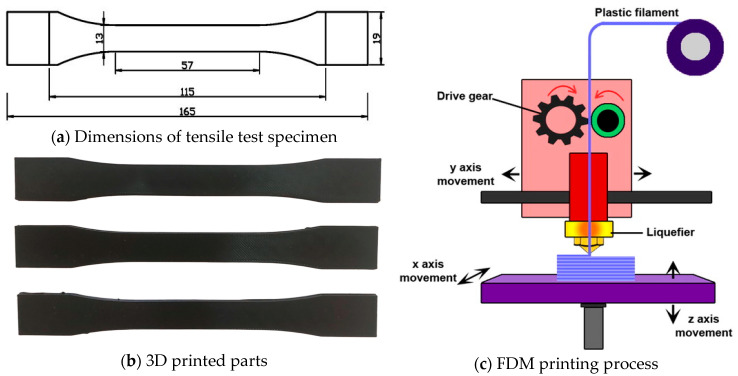
Designed specimen dimensions and FDM printing process.

In this study, all specimens were manufactured using the FDM method with a Zaxe Z1 Plus 3D printer (Zaxe Inc., Istanbul, Turkey), as illustrated in Figure 1c. This printer supports a range of engineering-grade thermoplastic materials and features a heated bed, a high-precision extrusion system, and compatibility with a 1.75 mm filament diameter, enabling the fabrication of mechanically robust and dimensionally accurate parts. The FDM process provides a layer-by-layer AM approach, which makes it ideal for producing complex geometries and functional prototypes with consistent quality [31]. The fabrication workflow began with the design of the tensile test specimens in accordance with the ASTM D638; Standard Test Method for Tensile Properties of Plastics. ASTM International: West Conshohocken, PA, USA, 2022. The dimensions of these specimens are detailed in Figure 1a. The 3D models were created using SolidWorks CAD software version 2019 and subsequently exported in standard tessellation language (STL) format. This format converts geometry into a mesh of triangular facets that can be interpreted by slicing software (xDesktop slicer software). The STL files were processed using slicing software to generate G-code instructions, which define crucial printing parameters such as extrusion paths, temperature settings, and layer height. These G-code files were then transferred to the 3D printer to execute the fabrication process.

All specimens were printed in the XY plane and subjected to loads in the Z direction during testing. The printing process was conducted under controlled conditions to ensure consistency and repeatability. As part of our design of experiments (DoE) methodology, several printing parameters were systematically varied to investigate their influence on the resulting mechanical performance. The variable parameters and their corresponding levels are listed in Table 1. While the selected variables were altered according to the experimental design, certain printing parameters were held constant throughout all fabrication runs to isolate the effects of the variables under study. These constant parameters included a nozzle diameter of 0.4 mm, a bed temperature of 70 °C, six contour lines to define the outer perimeters of the parts, and a raster width of 0.50 mm, which resulted in an overall wall thickness of 4 mm. These fixed parameters were chosen based on material recommendations and preliminary trials to ensure sufficient interlayer adhesion and surface quality. The FDM process was applied to the fabrication of all tensile test specimens using PA12-CF filament. Several example specimens produced during the fabrication process are presented in Figure 1b, which illustrates the consistency of the printed parts and their adherence to the original design specifications. The combination of optimized process parameters, high-performance material selection, and accurate machine calibration ensured the successful production of test samples suitable for mechanical testing and subsequent analysis.

In FDM technology, the selection and optimization of process parameters are critical to achieving high mechanical performance and surface quality in printed parts. The five key parameters that were identified for this study are as follows: layer thickness (LT), infill pattern (IP), nozzle temperature (NT), printing speed (PS), and infill density (ID). These parameters and their levels were selected based on a combination of the prior literature, manufacturer guidelines for PA12-CF, and initial trial runs [32,33]. Printing parameters are widely recognized for their significant influence on the structural and functional properties of 3D-printed components and collectively play a pivotal role in determining the quality, strength, and reliability of the final product. Each parameter was examined at three distinct levels, which are defined in Table 1, to capture a broad range of behaviors and potential interactions. For instance, the LT affects the bonding between layers and surface resolution; the PS influences not only the build time but also the interlayer adhesion due to thermal gradients; the NT determines the polymer flow and melting consistency; the ID governs the internal structure and, therefore, the stiffness and strength of the part; and the IP dictates how mechanical loads are distributed within the internal geometry of the printed object.

The Taguchi DoE methodology, specifically the L27 orthogonal array, was employed in this study to systematically investigate the influence of the selected parameters while minimizing the number of required experiments. The Taguchi method is a robust and widely used statistical approach that enables efficient experimentation by reducing the number of trials needed to analyze multi-factor systems. In a full-factorial design with five parameters at three levels each, a total of 243 (3^5^) experiments would be required. However, with the L27 array, only 27 experiments were conducted, which provided a substantial reduction in time, material usage, and cost without compromising the comprehensiveness of the analysis. The Taguchi approach not only facilitates the evaluation of individual parameter effects but also enables the identification of optimal parameter combinations that enhance the desired output characteristics. In this study, tensile strength and surface roughness were selected as the response variables that would be used to evaluate the mechanical and surface performance of the PA12-CF composite specimens. Through a systematic experimental design, this study intended to reveal which FDM parameters most strongly influence the performance of carbon fiber-reinforced 3D-printed components and determine their ideal settings. The experimental matrix and the corresponding results are presented in Table 2, and form the basis for further statistical and ML-based evaluations.

### 2.3. Experimental Tests

The performance of the FDM-printed PA12-CF composite specimens was evaluated through two primary experimental tests: tensile testing, which assessed mechanical strength, and surface roughness measurement, which characterized surface quality. These tests provide critical insights into the structural and functional capabilities of the printed parts, which are essential to their suitability for potential industrial and engineering applications. Tensile testing was performed to determine the tensile strength, which is defined as the maximum stress a material can withstand under uniaxial tension before failure. This property is particularly important for components subjected to mechanical loads in service, as it reflects the material’s ability to resist elongation and fracture. In this study, the tensile specimens were fabricated in accordance with the ASTM D638 standard, and the tests were carried out using a Shimadzu AG-X universal testing machine with a maximum load capacity of 50 kN. The testing procedure followed the standard protocol, using a crosshead speed of 1 mm/s, and the machine’s high precision (±0.1%) ensured accurate recording of force and elongation data. The resulting stress–strain curves provided valuable information on both the elastic modulus and plastic deformation behavior of the composite material.

In addition to their mechanical performance, the surface quality of the printed specimens was assessed through surface roughness measurements, which are crucial in applications where aesthetic appearance, frictional behavior, or interfacial bonding are of concern. Surface roughness was quantified using a Mitutoyo Surftest SJ-210 (Mitutoyo Corporation, Kawasaki, Japan) digital profilometer, a high-precision instrument capable of detecting micron-level height variations. Prior to measurement, the device was calibrated to ensure accurate results. During the test, the stylus was carefully moved across the designated surface of each sample to detect topographical variations. The profilometer automatically calculated the average surface roughness (Ra) value, which is a standard parameter representing the arithmetic mean of surface deviations measured in micrometers (μm). Lower Ra values indicate smoother surfaces, which are generally desirable due to providing better mechanical performance and reduced wear. To improve the reliability of the results, each surface was measured three times, and the average Ra value was recorded for analysis. The experimental results obtained from the tensile strength and surface roughness tests are presented in Table 2. These results serve as the foundation for the subsequent ML-based modeling and analysis conducted in this study to investigate the effects of different printing parameters on the resulting composite part quality.

### 2.4. Artificial Neural Network Method

**Figure 2:**
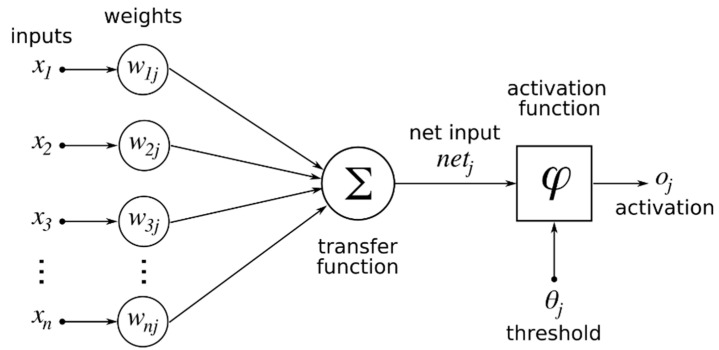
Structure of the artificial neural network model.

ANNs are a class of ML models inspired by the structure and functionality of the human brain. These models consist of interconnected layers of artificial neurons that are capable of learning complex and nonlinear relationships in data through iterative training processes [34,35]. Due to their high adaptability and predictive power, ANNs have become increasingly popular in engineering applications, particularly in areas such as data-driven modeling, optimization, and control. In the context of AM, where multiple process parameters interact in nonlinear and often unpredictable ways, ANNs offer a powerful solution for modeling and predicting the outcomes of production processes. Traditional analytical or statistical modeling techniques often fall short in capturing the intricate dependencies between variables such as the LT, IP, NT, PS, and ID. In contrast, ANNs can effectively learn from experimental data to uncover hidden patterns and provide accurate predictions, significantly reducing the time, effort, and cost associated with physical testing [36]. An ANN typically consists of three main components (Figure 2): an input layer, one or more hidden layers, and an output layer [37,38]. The input layer receives the initial data, which corresponds to the independent variables. The hidden layers process the inputs through weighted connections and apply nonlinear activation functions, allowing the network to capture complex relationships. The output layer then produces the final predictions—in this case, tensile strength and surface roughness.

The training of an ANN involves minimizing a predefined loss function, such as the mean squared error (MSE) for regression tasks, using optimization algorithms like the stochastic gradient descent (SGD) or Levenberg–Marquardt (TRAINLM) algorithms. During training, the network adjusts the weights of neuron connections using the backpropagation algorithm, which propagates the error between the predicted and actual values backward through the network and updates the weights accordingly. This learning process continues until the network reaches a predefined level of accuracy or a maximum number of iterations (epochs). In this study, the ANN model architecture included an input layer with five neurons, three hidden layers with 30 neurons each, and a final output layer with two neurons that represented the predicted tensile strength and surface roughness, respectively. The training process was conducted using the TRAINLM to achieve efficient convergence, and the gradient descent (LEARNGD) function was employed for weight adaptation. A total of 900 epochs were used to train the network, with the MSE being used as the performance function. By leveraging the capabilities of the constructed ANN, this study successfully predicted the material behavior of FDM-printed PA12-CF composite specimens under varying process conditions. The ANN not only modeled the nonlinear interactions between parameters but also provided insights into optimal production configurations. Overall, the use of ANNs in this study highlights their effectiveness for and versatility in predictive modeling in additive manufacturing, offering a robust tool for enhancing process efficiency and part quality.

## 3. Results and Discussion

### 3.1. Taguchi Design Results

In this section, the outcomes of the experimental design based on the Taguchi method are discussed. The experimental setup was created using the L27 orthogonal array, allowing for the systematic investigation of five key FDM process parameters—LT, IP, NT, PS, and ID, each at three levels. Instead of conducting a full factorial design that would require 243 experiments, the Taguchi method enabled the completion of this study with only 27 trials, significantly reducing the time and resource consumption while still capturing the essential effects of each variable (Table 2). Known for enhancing quality while minimizing cost and variability, the Taguchi method enables robust optimization by focusing on signal/noise (S/N) ratios. In this study, the tensile strength and surface roughness were selected as the performance metrics for FDM-printed PA12-CF composite specimens. Each sample was tested three times, and the average value was recorded to ensure the reliability of the data. To evaluate the effects of each parameter and determine the optimal combination, S/N ratios were calculated using the “larger is better” criterion for tensile strength and the “smaller is better” criterion for surface roughness. This dual-objective approach allowed for the identification of parameter settings that maximize the mechanical strength while minimizing surface irregularities.

The results revealed considerable variation in both response variables. The tensile strength values ranged from 5.24 MPa to 69.65 MPa, while the surface roughness varied between 9.18 µm and 18.79 µm. The highest tensile strength was achieved in Experiment 15, which incorporated a “moderate” LT, “honeycomb” IP, “high” NT, “low” PS, and “high” ID. This configuration proved to be highly effective in enhancing the material’s mechanical performance. The lowest tensile strength and highest surface roughness were recorded in Experiment 22, which involved a thick layer, low NT, sparse internal structure, and suboptimal material deposition. These findings suggest that improper parameter selection can drastically diminish both the strength and surface quality of printed parts. The strong correlation between high S/N ratios and superior mechanical properties confirms the robustness of the optimized parameter settings. Conversely, lower S/N ratios were associated with greater variability and weaker structural performance, indicating less desirable process conditions. Overall, the Taguchi method proved to be an efficient and reliable tool for identifying critical process variables and optimizing them to improve additive manufacturing outcomes. Furthermore, the experimental results obtained from this phase served as a valuable dataset for the ANN modeling conducted in the subsequent stages of this study.

**Figure 3:**
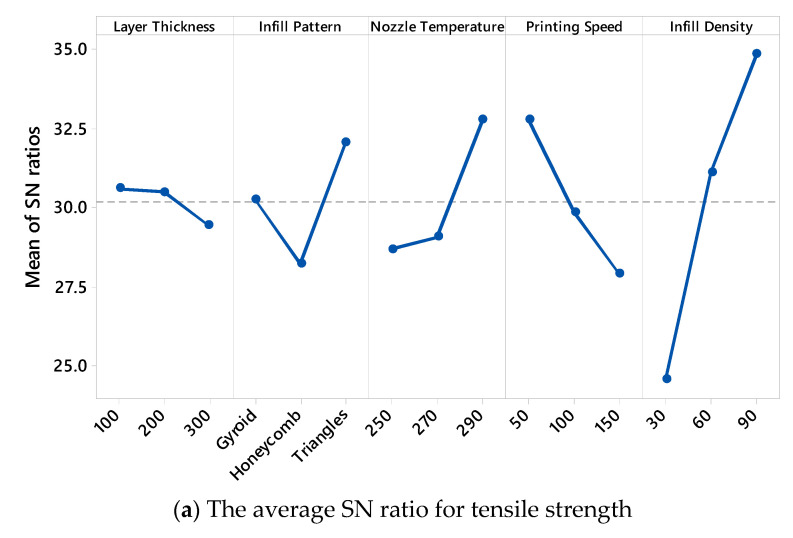

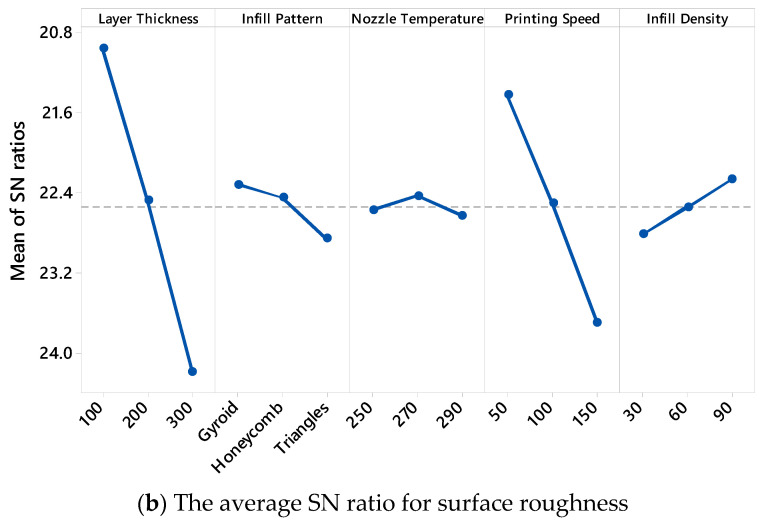
Main effect plots of SN ratios for tensile strength and surface roughness.

Figure 3 presents the main effect plots of the S/N ratios for both the tensile strength and surface roughness, offering valuable insights into how each FDM printing parameter influences the mechanical and surface properties of PA12-CF composites. The ID has a pronounced positive effect on the tensile strength (Figure 3a). As the ID increases from 30% to 90%, the mean S/N ratio rises significantly, with the highest value being observed at 90%. This clearly indicates that densely filled parts provide better structural integrity and strength. Similarly, the LT plays a crucial role; as the LT increases from 100 µm to 300 µm, a gradual decline in the S/N ratio is observed. This suggests that thinner layers enhance the bonding between the layers and contribute to higher mechanical performance, which aligns with the results of previous studies in the field. The PS also exhibits a notable influence—higher speeds result in a considerable decrease in the S/N ratio for the tensile strength. This trend implies that lower speeds allow for better material deposition and adhesion, ultimately improving the tensile response of the printed parts. In contrast, the effect of the NT is less pronounced but still noticeable. A moderate increase in the S/N ratio is seen as the temperature rises from 250 °C to 290 °C, suggesting that higher temperatures may slightly improve interlayer bonding, although the effect is not as dominant as that of the other parameters. Among the different IPs, the triangles pattern yields the highest S/N ratio, indicating superior mechanical performance compared to the gyroid and honeycomb patterns. Figure 3b illustrates the main effects of the variables on the surface roughness, where the goal was to minimize the Ra values, which corresponds to higher S/N ratios under the “smaller is better” criterion. Among the parameters, the LT has the most substantial impact. As the LT increases, the S/N ratio decreases dramatically, confirming that finer layers result in smoother surfaces. The PS, again, shows a similar trend, where higher speeds lead to rougher surfaces and thus lower S/N values. The ID also affects the surface quality, albeit with a smaller slope; higher densities tend to produce slightly smoother surfaces. Interestingly, the influence of the NT and IP on the surface roughness appears relatively limited, with only minor variations in the S/N ratios being observed across their levels. Overall, the analysis of the S/N ratio trends reveals that optimal tensile strength and minimal surface roughness can be achieved using a combination of “100 µm” LT, “triangles” IP, “290 °C” NT, “50 mm/s” PS, and “90%” ID. These observations align closely with earlier findings and emphasize the importance of fine-tuning process parameters in FDM to achieve enhanced mechanical performance and surface quality in composite materials.

### 3.2. Surface Plot Results

**Figure 4:**
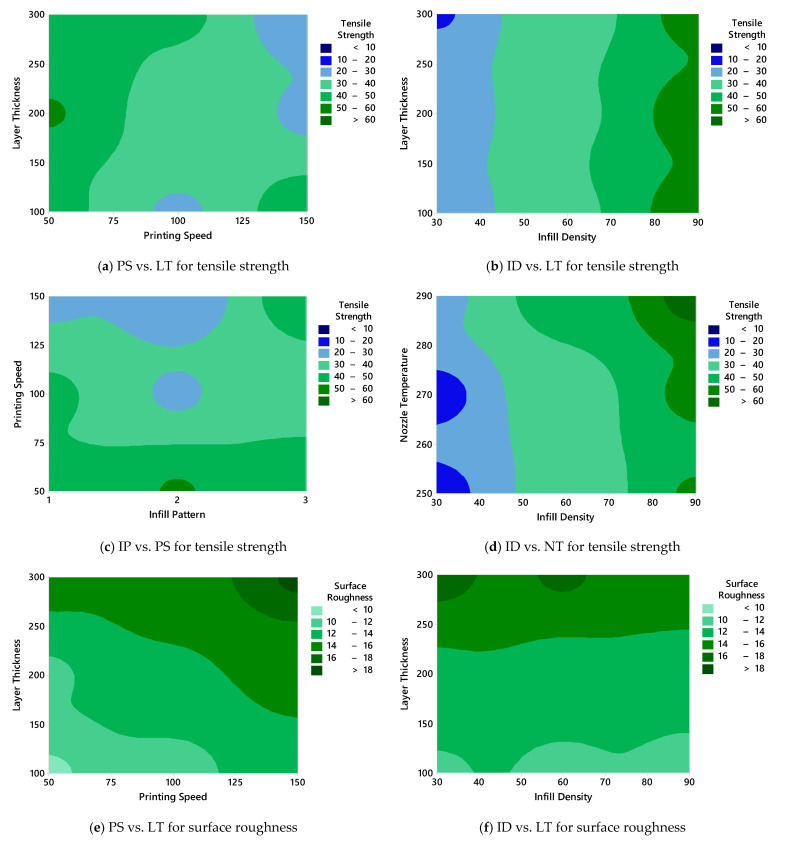

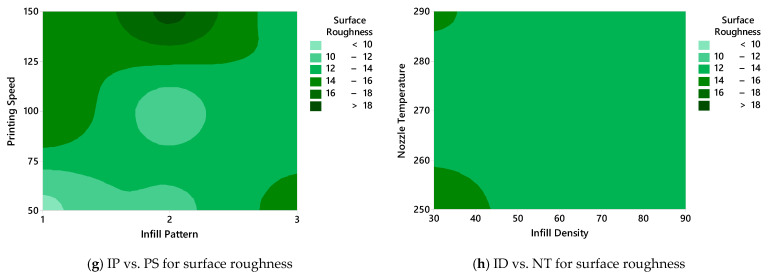
Surface plots of tensile strength and surface roughness with printing parameters.

In this section, surface plots are employed to visualize the interaction effects of FDM process parameters on the tensile strength and surface roughness of PA12-CF composite specimens. The surface plots presented in Figure 4 provide a comprehensive understanding of how combinations of two parameters simultaneously influence the output responses. This visual approach complements the main effect analysis by revealing synergistic or antagonistic interactions between the variables. Figure 4a illustrates the effect of the relationship between the PS and LT on the tensile strength. The highest strength values (>60 MPa) are observed when low to moderate PSs (50–75 mm/s) are combined with thinner layers (100–200 µm). As the LT increases toward 300 µm or the PS exceeds 100 mm/s, the tensile strength decreases significantly, indicating that rapid printing and thicker layers compromise the interlayer bonding and structural integrity. Figure 4b explores the combined effect of the ID and LT. A clear trend emerges, indicating that the tensile strength improves as the ID increases from 30% to 90%, regardless of the LT. However, the most favorable performance is achieved when a high ID is paired with thinner layers, reinforcing the conclusion that dense internal structures and a fine layer resolution contribute synergistically to mechanical enhancement. Figure 4c shows the interaction between the IP and PS. While triangle patterns generally yield higher strength values, the effect becomes more prominent at lower PSs. At higher speeds, the influence of the pattern type diminishes, and the tensile strength tends to decline across all patterns, suggesting that optimal deposition conditions are crucial to realizing the benefits of specific infill geometries. Figure 4d examines the effect of the NT and ID. Higher NTs (270–290 °C) combined with a higher ID (70–90%) results in significantly improved tensile strength (>60 MPa). In contrast, the combination of a low temperature and low infill leads to poor mechanical performance. This confirms that elevated temperatures enhance polymer flow and interlayer adhesion, especially when more material is deposited internally.

The corresponding surface plots for the surface roughness are shown in Figure 4e–h. Figure 4e reveals that increasing both the PS and LT contributes to higher roughness values (>16 µm). The smoothest surfaces (<12 µm) are achieved at low speeds and thin layers, which aligns with the known surface finish benefits of using finer resolutions and slower deposition rates. Figure 4f displays the interaction between the ID and LT. Interestingly, the roughness does not vary drastically with the ID but increases steadily with the LT. This suggests that, unlike the tensile strength, the surface finish is less sensitive to the internal structure of the material and more influenced by the vertical resolution. Figure 4g investigates the IP and PS. As for the tensile strength, lower roughness is found at slower PSs across all IPs. However, the pattern type introduces localized variations, with some combinations yielding rougher or smoother results. This indicates that the geometric complexity of the internal structure may subtly affect the outer surface formation under varying speeds. Lastly, Figure 4h presents the interaction between the ID and NT. In this case, the surface roughness remains relatively stable across all parameter combinations, with only minor differences being observed. This implies that these two variables have a limited combined influence on the Ra compared to others like the LT or PS. In summary, the surface plots clearly demonstrate that the optimal tensile strength and surface quality can be achieved through the combined tuning of key parameters. Specifically, a low LT (100 µm), low PS (50 mm/s), high ID (90%), and elevated NT (290 °C) emerge as the most favorable settings. These insights are critical for establishing robust processing windows in the FDM-based production of high-performance composite parts.

### 3.3. Analysis of Variance

ANOVA was conducted to statistically evaluate the influence of individual process parameters on the tensile strength and surface roughness of PA12-CF composite samples. The results, as summarized in Table 3, reveal that all five input parameters have statistically significant effects on both response variables, with *p*-values of less than 0.05 in all cases. This confirms that variations in these factors meaningfully impact the output performance in the FDM process. For the tensile strength, the most influential factor was the ID, with an adjusted sum of squares (Adj SS) of 6044.07 and a remarkably high F-value of 2901.21 indicating its dominant role in determining the resulting mechanical strength. This was followed by the PS (F = 512.04) and NT (F = 478.60), both of which also demonstrated substantial impacts. The IP and LT, though statistically significant, showed relatively lower F-values of 230.43 and 18.32, respectively, which suggests that they play a secondary role in determining the tensile performance. The coefficient of determination (R^2^) for the model was 98.16%, indicating that the model explains a very high proportion of the variability in tensile strength. In the case of surface roughness, the LT emerged as the most critical parameter, with an exceptionally high F-value of 2936.59 highlighting its overwhelming influence on the surface quality. This is consistent with the well-established understanding that finer layer resolutions lead to smoother surfaces. The PS also had a significant effect (F = 1410.51), reinforcing the notion that slower deposition improves the surface finish. While the IP, NT, and ID were also statistically significant (with *p*-values of 0.000), their F-values were considerably lower, indicating a more limited effect on the Ra. The R^2^ value of 97.32% confirms the model’s robustness in explaining variations in surface roughness. Overall, the ANOVA findings validate the trends observed in the main effect and surface plots. The results underscore the importance of optimizing the ID, NT, and PS to maximize the tensile strength, while emphasizing the LT and PS as the dominant factors in reducing surface roughness. These insights provide a statistically sound foundation for process optimization in the FDM-based production of fiber-reinforced polymer composites.

**Figure 5:**
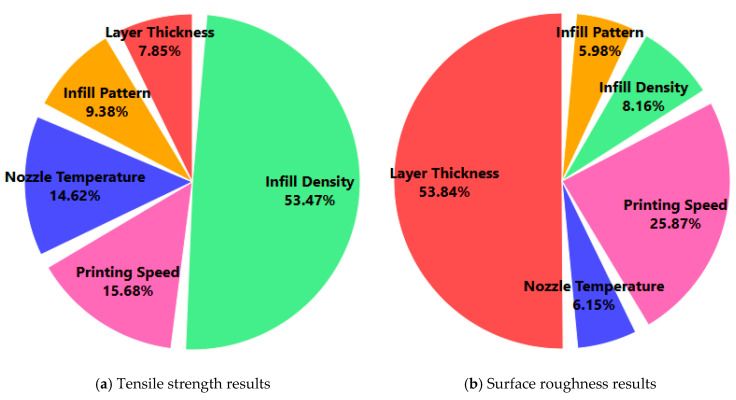
Contribution rates of printing parameters.

Figure 5 illustrates the contribution rates of the five printing parameters on the tensile strength and surface roughness using pie charts derived from the adjusted sum of squares (Adj SS) values reported in the ANOVA analysis. These visualizations provide a clear representation of the relative influence of each parameter on the two primary output responses of the FDM process. For the tensile strength, the most dominant factor is the ID, which accounts for 53.47% of the total variation. This substantial contribution highlights the critical role of the internal material distribution in determining the mechanical integrity of printed parts. Following this, the PS contributes 15.68%, and the NT contributes 14.62%, which indicates moderate that both exert but important effects. These parameters influence the bonding quality and material flow, which in turn affect the interlayer adhesion and tensile performance. The IP and LT have smaller contributions of 9.38% and 7.85%, respectively, which suggests that, while they are statistically significant, their influence on the tensile strength is relatively limited compared to that of the other parameters. In contrast, the contribution chart for the surface roughness reveals a different hierarchy. Here, the LT emerges as the most impactful factor, contributing 53.84% of the total variation. This result aligns with the expectation that finer layer resolutions yield smoother surface finishes. The PS follows with a notable contribution of 25.87%, which emphasizes that slower printing improves the surface quality by allowing more accurate material deposition. The ID (8.16%), NT (6.15%), and IP (5.98%) contribute less significantly, which indicates that the surface roughness is primarily governed by geometric and deposition rate-related factors rather than internal structural parameters. In summary, the contribution rates depicted in Figure 5 confirm the distinct influence patterns of the process parameters on different quality metrics. While the ID plays a dominant role in enhancing the tensile strength, the LT is the key determinant of the surface roughness. These findings are essential to prioritizing different parameter optimization strategies depending on the target performance—whether it requires better mechanical strength or surface quality—in FDM-based AM.

### 3.4. Artificial Neural Network Results

Predicting outcomes in advance is a critical step toward achieving reliable and efficient results in AM [39,40]. In the context of production with FDM, anticipating the effects of printing parameters on the resulting part quality can lead to significant time and cost savings through the optimized use of resources. In recent years, AI techniques have gained increasing prominence in AM research, particularly ANNs, which are inspired by the biological structure of the human brain [41,42]. These systems can learn from historical data and generalize that learning to predict future events, which makes them highly suitable for modeling the complex, nonlinear relationships inherent in AM processes. In this study, an ANN model was developed to predict the tensile strength and surface quality of test specimens manufactured from PA12-CF using the FDM technique. The ANN was trained to model the relationships between five key process parameters and the resulting performance metrics. A total of 54 data points (derived from two response variables across 27 experimental combinations) were prepared and randomly split into three subsets: 75% for training, 10% for validation, and 15% for testing. This distribution was selected to ensure sufficient learning while minimizing overfitting and improving the model’s generalization capability. The ANN was implemented using a feedforward neural network architecture within the MATLAB (software version 2019b) environment. A single hidden layer composed of 10 neurons was used, and the sigmoid activation function was applied in both the hidden and output layers to allow for the nonlinear mapping of inputs to outputs. The model was trained using the Levenberg–Marquardt algorithm, which is known for its efficiency and fast convergence that make it particularly suitable for small- to medium-sized datasets with complex dependencies.

The predictive performance of the ANN model was evaluated using six widely accepted error metrics: the mean squared error (MSE), mean absolute error (MAE), root mean square error (RMSE), mean absolute percentage error (MAPE), coefficient of determination (R^2^), and correlation coefficient (r). These statistical indicators offer a comprehensive evaluation that covers both absolute and relative prediction errors, as well as the degree of linear correlation between predicted and actual values. Among these, the R^2^ value is particularly important, as it reflects how well a model can explain the variance in observed data. An R^2^ value close to 1 indicates excellent predictive accuracy. In addition to the ANN model, three other ML algorithms were employed for comparative analysis: SVR, RFR, and XGBoost models. These models were trained on the same dataset and evaluated using identical performance metrics. This comparative study aimed to benchmark the ANN’s prediction capability against other widely used data-driven modeling approaches. The ANN model consistently outperformed the SVM, RFR, and XGBoost models by obtaining lower error values and higher correlation, which confirmed its superior ability to capture the complex interactions among process parameters in the FDM process.

### 3.5. ANN of Tensile Strength

**Figure 6:**
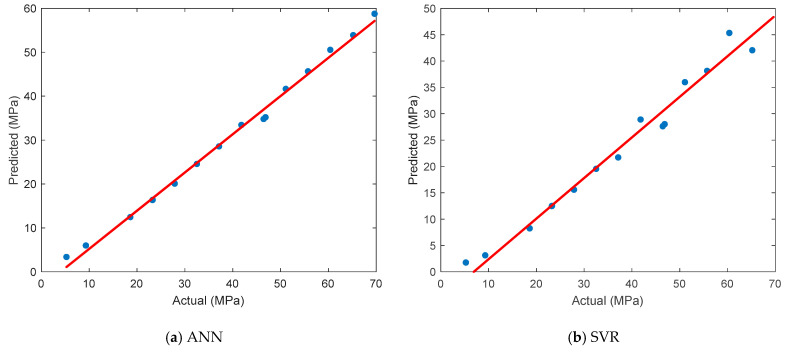

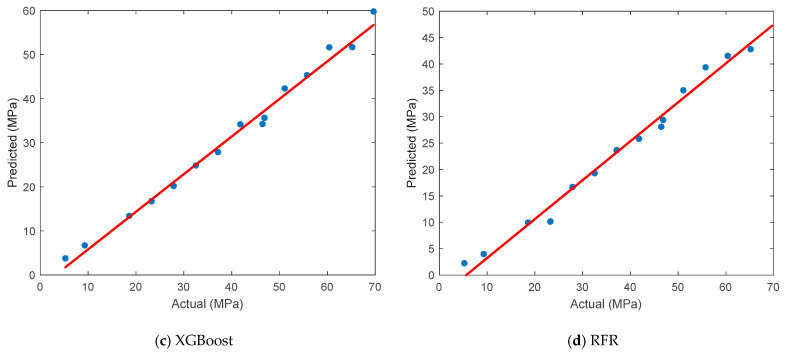
Comparison of the actual and predicted results for tensile strength.

In this section, the prediction performance of the ANN model for tensile strength is evaluated and compared with that of three other widely used machine learning algorithms. The comparison is visualized in Figure 6, which presents scatter plots that illustrate the relationship between the actual and predicted tensile strength values for each model. As shown in Figure 6a, the ANN demonstrated excellent agreement between the experimental and predicted values. The data points are tightly clustered along the ideal diagonal line (red line), indicating that the ANN successfully captured the complex nonlinear relationships among the FDM process parameters and tensile strength. Due to its robust learning capability, the ANN achieved the highest prediction accuracy compared to the other models. Figure 6b displays the performance of the SVR model, which exhibited wider dispersion and a tendency to underestimate higher tensile strength values. This behavior resulted in higher prediction errors and a weaker correlation with the actual measurements. In Figure 6c, the XGBoost model shows relatively strong predictive performance, with predicted values that closely follow the actual data trend. It ranked second overall and was particularly effective in modeling mid-range tensile strength values, although some deviations were observed at the extremes. Figure 6d presents the prediction accuracy of the RFR model. Although it performed better than the SVR model, its predictions were more scattered compared to the ANN and XGBoost models, especially at high tensile strength levels.

The effectiveness of various ML models in predicting the tensile strength of PA12-CF composite parts produced by FDM was evaluated by comparing four different approaches using several error performance metrics. The detailed results are presented in Table 4. The ANN model consistently achieved the best results across all performance indicators. It attained the lowest MSE (0.7938), MAE (2.6581), and RMSE (0.8910), along with the lowest MAPE (11.35%), indicating a high prediction accuracy and minimal deviation from the actual values. Moreover, its R^2^ value of 0.9912 and correlation coefficient (r) of 0.9956 confirm a very strong linear relationship between the predicted and actual tensile strength values. The XGBoost model followed as the second-best performer, outperforming the RFR and SVR models, particularly in terms of its MAPE and MAE values. Although its RMSE (1.1312) was slightly higher than that of the ANN, it maintained a high R^2^ of 0.9875, which suggests a reliable generalization capability. The RFR model ranked third, showing good correlation (r = 0.9931) but slightly higher prediction errors, particularly in terms of its MAE and MAPE. While the RFR model effectively captured the overall trend, it demonstrated less precision at the extremes of the tensile strength range. The SVR model yielded the lowest performance among the four models. With the highest error values (MSE = 1.7538, MAE = 4.5836, RMSE = 1.3243, MAPE = 25.37%) and the lowest R^2^ (0.9795), it struggled to accurately predict the tensile strength, especially for higher values. Overall, these results confirm that the ANN model is the most effective tool for predicting tensile strength among those tested in this study, thanks to its ability to capture complex nonlinear relationships within the dataset. Its superior performance compared to other models highlights its potential as a reliable decision-support tool in process optimization for FDM-based production using PA12-CF composites.

### 3.6. ANN of Surface Roughness

**Figure 7:**
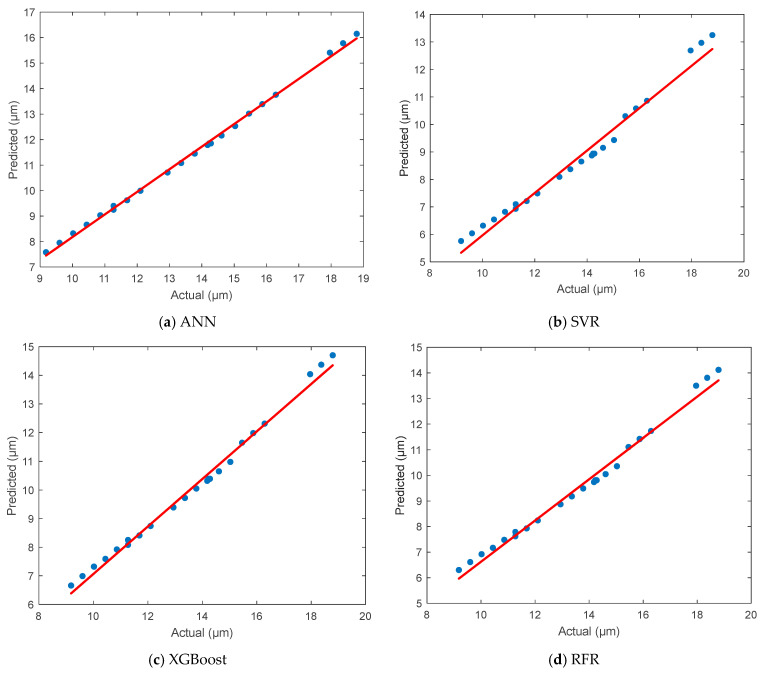
Comparison of the actual and predicted results for surface roughness.

In this section, the prediction performance of the ANN model for surface roughness is evaluated and compared with three other ML algorithms. The scatter plots in Figure 7 depict the correlation between the actual and predicted values for each model. In Figure 7a, the ANN demonstrates an outstanding prediction capability, with the data points being tightly aligned along the ideal red line. This indicates a very strong agreement between the actual and predicted surface roughness values. The distribution of points reveals minimal deviation, suggesting that the ANN captured the underlying nonlinear relationships between the process parameters and surface quality with high accuracy. Figure 7b shows the performance of the SVR model, which presents noticeable dispersion, particularly in the mid- and high-value ranges of surface roughness. The predicted values tend to be underestimations the actual values, especially at the upper end, indicating a limitation in the model’s generalization ability for this dataset. In Figure 7c, it can be seen that the XGBoost model provided relatively accurate predictions, with many points closely following the diagonal reference line. While not as precise as the ANN, it still captures the trend effectively and ranks as the second-best performer, especially in the mid-range of surface roughness values. Figure 7d presents the results of the RFR model, which performs better than SVR and shows moderate alignment with the ideal trend line. However, some scattering is observed at both ends of the roughness range, indicating a slight loss in prediction precision compared to the ANN and XGBoost models.

The prediction accuracy of all models was evaluated using statistical performance metrics, including the MSE, MAE, RMSE, MAPE, R^2^, and r. The detailed results are presented below in Table 5. The ANN outperformed all other models, achieving the lowest MSE (0.3789) and MAPE (2.18%), which indicates a high accuracy and generalization capability. Its R^2^ value of 0.9945 and its correlation coefficient of 0.9972 further confirm the model’s excellent predictive power. The XGBoost model ranked second, with a low number of errors and high correlation values, making it a reliable alternative to ANNs. The RFR model followed closely behind, with slightly higher error rates but a predictive performance that was still acceptable. Finally, the SVR model showed the poorest performance, with the highest error values and the weakest correlation with the actual surface roughness measurements. In summary, the ANN model proved to be the most effective and accurate approach for predicting surface roughness in FDM processes using PA12-CF material. Its superior performance compared to the other models reinforces its suitability for use in surface quality optimization and intelligent process control in AM.

### 3.7. Validation Test Results of ANN

A separate validation dataset, which was not involved in the training, was employed to examine the generalization capability and practical relevance of the ANN. The predicted results of the ANN for both tensile strength and surface roughness were compared with the actual experimental values and with the predictions obtained from three other machine learning algorithms: RFR, XGBoost, and SVR models. The results reveal that the ANN model demonstrates a consistently high level of accuracy across both output parameters (Table 6). For the tensile strength predictions, the ANN model yielded values that were remarkably close to the actual measurements. The absolute differences were minimal, with a maximum absolute error of only 0.06 MPa, corresponding to a percentage deviation of approximately 0.22% in the worst case. This indicates that the ANN model successfully captured the underlying patterns in the data and generalized them accurately to unseen input combinations. Compared to the ANN, the RFR and XGBoost models also performed reasonably well but exhibited slightly larger deviations. The SVR model, on the other hand, consistently underestimated the tensile strength across all validation samples, with a maximum deviation exceeding 1.2 MPa. This underestimation tendency confirms the limitations of SVR models in modeling the complex nonlinear behavior of the tensile strength response in FDM-produced PA12-CF parts. A similar trend was observed in the prediction of surface roughness. The ANN model achieved exceptional results, with the predicted values matching the actual measurements almost exactly. The maximum deviation between the predicted and actual surface roughness was just 0.03 µm, which corresponds to a percentage error of only 0.20%. This level of precision demonstrates the ANN model’s excellent capability in modeling surface quality characteristics based on FDM process parameters. The XGBoost and RFR models also provided acceptable results, with slightly larger deviations, especially for higher roughness values. The SVR model again showed the weakest performance among the models, with errors reaching up to 0.38 µm and percentage deviations exceeding 2.5%, which indicates its limited reliability in capturing the surface roughness behavior of the system. Overall, the validation test results confirm that the ANN model possesses superior accuracy and robustness in predicting both the mechanical and surface properties of 3D-printed PA12-CF components. Its ability to generalize well with new data without overfitting makes it a powerful and reliable tool for process control and quality prediction in additive manufacturing applications. The consistent superiority of the ANN model across different output parameters highlights its potential for integration into intelligent decision-making systems for the real-time optimization of FDM processes.

### 3.8. Discussion

This study explored the effects of key FDM process parameters on the tensile strength and surface roughness of carbon fiber-reinforced PA12 composites, which was supported by experimental data and machine learning-based prediction models. The results revealed that the nozzle temperature, infill density, and printing speed significantly influence the resulting tensile strength, while the layer thickness and infill pattern are more closely associated with the surface roughness. The discussion below addresses several key considerations that were raised in relation to the findings and their broader implications. First, regarding the relevance of the selected process parameters to industrial production, it is important to note that the parameter ranges utilized in this study were determined based on a combination of manufacturer guidelines, reports from the literature, and settings that are commonly used in actual FDM manufacturing environments. Parameters such as the nozzle temperature (270–290 °C), infill density (60–100%), and printing speed (40–60 mm/s) are representative of realistic production conditions for high-performance carbon fiber-reinforced thermoplastics. Therefore, the findings are not only experimentally valid but also practically applicable to industrial-scale additive manufacturing processes. Second, this study places particular emphasis on surface roughness due to its practical and mechanical significance in real-world applications. Although the surface roughness does not directly determine the tensile strength under quasi-static loading, it plays a critical role in the fatigue life, friction behavior, and bonding performance of assembled components. Rough surfaces may act as stress risers or initiation sites for microcracks, especially in composite structures. Therefore, analyzing the surface roughness alongside the tensile strength provides a more complete understanding of the mechanical integrity and functional performance of printed parts. Third, in response to the question of the applicability of the proposed method to lignocellulosic (natural fiber) composites, it is acknowledged that such materials exhibit higher variability due to their natural origin. However, the use of ANNs and other ML algorithms is particularly well-suited to handling complex, nonlinear, and variable datasets. With sufficient experimental data reflecting the variability of fiber sources, volume fractions, and moisture content, the predictive framework presented in this study could be adapted to forecast the performance of bio-based composites. This would be especially valuable in advancing the use of different materials in AM, where process–property relationships are often less predictable. From an industrial perspective, the integration of data-driven models into additive manufacturing workflows can support real-time process optimization, quality control, and material selection strategies. The dual-output ANN developed in this research offers a foundation for the construction of intelligent manufacturing systems that can predict and adjust mechanical and surface properties before production is completed. Such capabilities are critical in sectors like the automotive, aerospace, robotics, and functional prototyping sectors, where high performance and reliability are essential. As for future work, the methodology established in this study can be extended in several directions. These include incorporating other material systems, such as hybrid or natural fiber composites, expanding the output metrics to include properties like the impact strength or fatigue resistance, and developing closed-loop control systems where real-time sensor data are fed into predictive models for adaptive process adjustments. Additionally, the creation of a broader materials database could enhance the model’s generalizability and enable the use of automated design-for-manufacturing tools in FDM applications. In conclusion, this study not only provides insights into the relationship between FDM parameters and part performance but also lays the groundwork for data-driven manufacturing strategies that are applicable to both synthetic and natural composite materials.

## 4. Conclusions

This study presents a comprehensive framework that combines experimental investigation, statistical analysis, and ML modeling to analyze and predict the tensile strength and surface roughness of PA12-CF composite materials manufactured using FDM. The experimental setup was designed using a Taguchi L27 orthogonal array, which enabled the efficient exploration of five critical printing parameters. The Taguchi analysis and ANOVA revealed that the ID was the most influential parameter for tensile strength, contributing 53.47% to the observed variation, followed by the PS and NT. For surface roughness, the LT had the highest contribution at 53.84%, confirming that finer layers result in smoother surfaces. An ANN model was developed to predict both the tensile strength and surface roughness. A comparative analysis was conducted with three other ML models. The ANN model consistently outperformed the others in all evaluation metrics. For tensile strength, the ANN achieved the lowest MSE of 0.7938, MAPE of 11.35%, and R^2^ of 0.9912, indicating a strong predictive capability. For surface roughness, the ANN model demonstrated near-perfect accuracy with an MSE of 0.3789, a MAPE of 2.18%, and a r of 0.9972. The validation tests using unseen experimental data further confirmed the model’s generalization power, with the maximum percentage errors remaining below 0.41% for both output variables. These results establish ANNs as a robust and highly reliable modeling tool for FDM-printed composite materials. The integration of Taguchi-based design, ANOVA, and ANN modeling in this study presents a hybrid methodology that combines experimental precision with intelligent prediction. This approach enables data-driven process optimization in additive manufacturing, particularly in the AM of fiber-reinforced thermoplastics with complex, nonlinear behavior. The study highlights the strength of ML, especially ANNs, not only in predicting mechanical properties but also in estimating surface quality—an often overlooked aspect in previous research. The proposed framework aligns with Industry 4.0 objectives, offering potential for use in real-time process control and digital twin applications. Future work may involve expanding the model to predict additional properties, integrating real-time sensor data, and applying the methodology to other reinforcement types to obtain broader industrial relevance.

## Figures and Tables

**Table 1:** Printing parameters used in the experimental process.

Parameters	Symbol	Units	Level 1	Level 2	Level 3
Layer Thickness	LT	µm	100	200	300
Infill Pattern	IP	-	Gyroid	Honeycomb	Triangles
Nozzle Temperature	NT	°C	250	270	290
Printing Speed	PS	mm/s	50	100	150
Infill Density	ID	%	30	60	90

**Table 2:** Experimental design results for PA12-CF composites.

	Printing Parameters					Experimental Results	
No	Layer Thickness	Infill Pattern	Nozzle Temperature	Printing Speed	Infill Density	Tensile Strength	Surface Roughness
1	100	Gyroid	250	50	30	23.23	10.02
2	100	Gyroid	250	50	60	41.81	9.60
3	100	Gyroid	250	50	90	60.39	9.18
4	100	Honeycomb	270	100	30	9.29	11.27
5	100	Honeycomb	270	100	60	27.87	10.86
6	100	Honeycomb	270	100	90	46.85	10.44
7	100	Triangles	290	150	30	27.87	13.78
8	100	Triangles	290	150	60	46.45	13.36
9	100	Triangles	290	150	90	65.19	12.94
10	200	Gyroid	270	150	30	9.29	15.03
11	200	Gyroid	270	150	60	27.87	14.61
12	200	Gyroid	270	150	90	46.45	14.28
13	200	Honeycomb	290	50	30	32.51	12.10
14	200	Honeycomb	290	50	60	51.09	11.69
15	200	Honeycomb	290	50	90	69.65	11.27
16	200	Triangles	250	100	30	18.59	14.23
17	200	Triangles	250	100	60	37.13	13.78
18	200	Triangles	250	100	90	55.74	13.36
19	300	Gyroid	290	100	30	23.27	16.29
20	300	Gyroid	290	100	60	41.81	15.87
21	300	Gyroid	290	100	90	60.39	15.46
22	300	Honeycomb	250	150	30	5.24	18.79
23	300	Honeycomb	250	150	60	18.59	18.37
24	300	Honeycomb	250	150	90	37.13	17.96
25	300	Triangles	270	50	30	27.87	15.03
26	300	Triangles	270	50	60	46.45	14.61
27	300	Triangles	270	50	90	65.19	14.18

**Table 3:** ANOVA results for input-output parameters of PA12-CF composites.

Factors	Tensile Strength				Surface Roughness			
	Adj SS	Adj MS	F-Value	*p*-Value	Adj SS	Adj MS	F-Value	*p*-Value
Layer Thickness	38.17	19.09	18.32	0.000	114.20	57.1005	2936.59	0.000
Infill Pattern	480.06	240.03	230.43	0.000	1.383	6.917	35.572	0.000
Nozzle Temperature	997.06	498.53	478.60	0.000	1.389	6.944	35.714	0.000
Printing Speed	1066.73	533.37	512.04	0.000	54.853	27.4267	1410.51	0.003
Infill Density	6044.07	3022.03	2901.21	0.000	3.117	1.5583	80.143	0.000
Degree of Freedom	26				26			
R-squared	98.16%				97.32			

**Table 4:** Prediction results for tensile strength using different models.

Model	MSE	MAE	RMSE	MAPE (%)	R^2^	Correlation (r)
ANN	0.7938	2.6581	0.8910	11.3479	0.9912	0.9956
RFR	1.4923	3.9573	1.2216	23.4928	0.9862	0.9931
XGBoost	1.2795	3.1867	1.1312	19.8537	0.9875	0.9937
SVR	1.7538	4.5836	1.3243	25.3681	0.9795	0.9897

**Table 5:** Prediction results for surface roughness using different models.

Model	MSE	MAE	RMSE	MAPE (%)	R^2^	Correlation (r)
ANN	0.3789	0.8561	0.6153	2.1837	0.9945	0.9972
RFR	0.6739	1.1983	0.8209	6.1352	0.9897	0.9948
XGBoost	0.5138	0.9823	0.7168	4.7381	0.9915	0.9957
SVR	0.9736	1.3692	0.9867	7.5893	0.9813	0.9906

**Table 6:** Comparative validation results of models for tensile strength and surface roughness.

Output Parameters	No	Printing Parameters					Actual	ANN	RFR	XGBoost	SVR
		LT (µm)	IP (-)	NT (°C)	PS (mm/s)	ID (%)		Predicted	Predicted	Predicted	Predicted
Tensile Strength	1	200	Gyroid	250	150	30	18.59	18.55	18.45	18.48	18.21
	2	300	Triangles	290	150	60	60.39	60.32	59.91	60.15	59.15
	3	100	Honeycomb	290	150	90	55.74	55.68	55.12	55.55	54.62
	4	200	Gyroid	290	100	90	60.39	60.35	59.78	60.25	59.15
	5	300	Gyroid	250	100	60	27.87	27.83	27.45	27.71	27.32
Surface Roughness	1	200	Triangles	270	150	60	15.45	15.42	15.23	15.35	15.02
	2	300	Honeycomb	290	100	30	16.70	16.67	16.41	16.55	16.23
	3	100	Gyroid	270	50	90	8.35	8.34	8.23	8.31	8.12
	4	300	Triangles	250	150	90	18.37	18.33	18.15	18.25	17.98
	5	200	Honeycomb	250	100	60	14.20	14.17	13.95	14.12	13.82

## Data Availability

The original contributions presented in this study are included in the article. Further inquiries can be directed to the corresponding author.
